# Outpatient respiratory syncytial virus infections and novel preventive interventions

**DOI:** 10.1097/MOP.0000000000001323

**Published:** 2023-12-12

**Authors:** Sarah F. Hak, Roderick P. Venekamp, Joanne G. Wildenbeest, Louis J. Bont

**Affiliations:** a Department of Pediatric Infectious Diseases and Immunology, Wilhelmina Children's Hospital/University Medical Center Utrecht; b Department of General Practice & Nursing Science, Julius Center for Health Sciences and Primary Care, University Medical Center Utrecht, Utrecht University, Utrecht; c Respiratory Syncytial Virus NETwork (ReSViNET) Foundation, Zeist, The Netherlands

**Keywords:** maternal immunization, mAbs, outpatient, respiratory syncytial virus, vaccines

## Abstract

**Purpose of review:**

With interventions to prevent respiratory syncytial virus (RSV) infection within reach, this review aims to provide healthcare professionals with the latest information necessary to inform parents and assess the potential impact of RSV prevention on everyday practice. We address frequently asked questions for parental counseling.

**Recent findings:**

Numerous studies emphasize the major burden of RSV on young children, parents, healthcare and society. In the first year of life, about 14% of healthy term infants visit a doctor and 2% require hospitalization due to RSV. In older children (1--5 years), RSV infections and associated morbidity (wheeze, acute otitis media) are major drivers of outpatient visits. A novel maternal RSV vaccine and long-acting mAb can provide protection during infants’ first months of life. This maternal vaccine showed 70.9% efficacy against severe RSV infection within 150 days after birth; the mAb nirsevimab reduces medically attended RSV infections by 79.5% within 150 days after administration. Both gained regulatory approval in the USA (FDA) and Europe (EMA).

**Summary:**

Novel RSV immunizations hold promise to reduce the RSV burden in infants, with substantial impact on everyday practice. Tailored parental guidance will be instrumental for successful implementation. Awaiting pediatric vaccines, RSV infections beyond infancy will still pose a significant outpatient burden.

## INTRODUCTION

In infants and young children, respiratory syncytial virus (RSV) is the leading cause of acute lower respiratory tract infections (LRTIs) and a major driver of doctor visits and hospitalizations, posing a substantial medical and societal burden [1^▪▪^,^2^]. By the age of 2 years, nearly all children experienced at least one RSV infection [3].

Until recently, the only preventive strategy against RSV was passive immunization with palivizumab, a humanized monoclonal RSV antibody. However, due to its high costs and need for monthly injections, its use is preserved for high-risk groups. Recently, a maternal RSV vaccine and a long-acting mAb for all infants have been approved in the USA and Europe [4–7]. Both passive immunizations offer protection against severe RSV infection during the first months of life, thereby minimalizing the infants’ window of critical vulnerability. Either one or both products are likely to be introduced in national immunization programs across the world. 

**Box 1:**
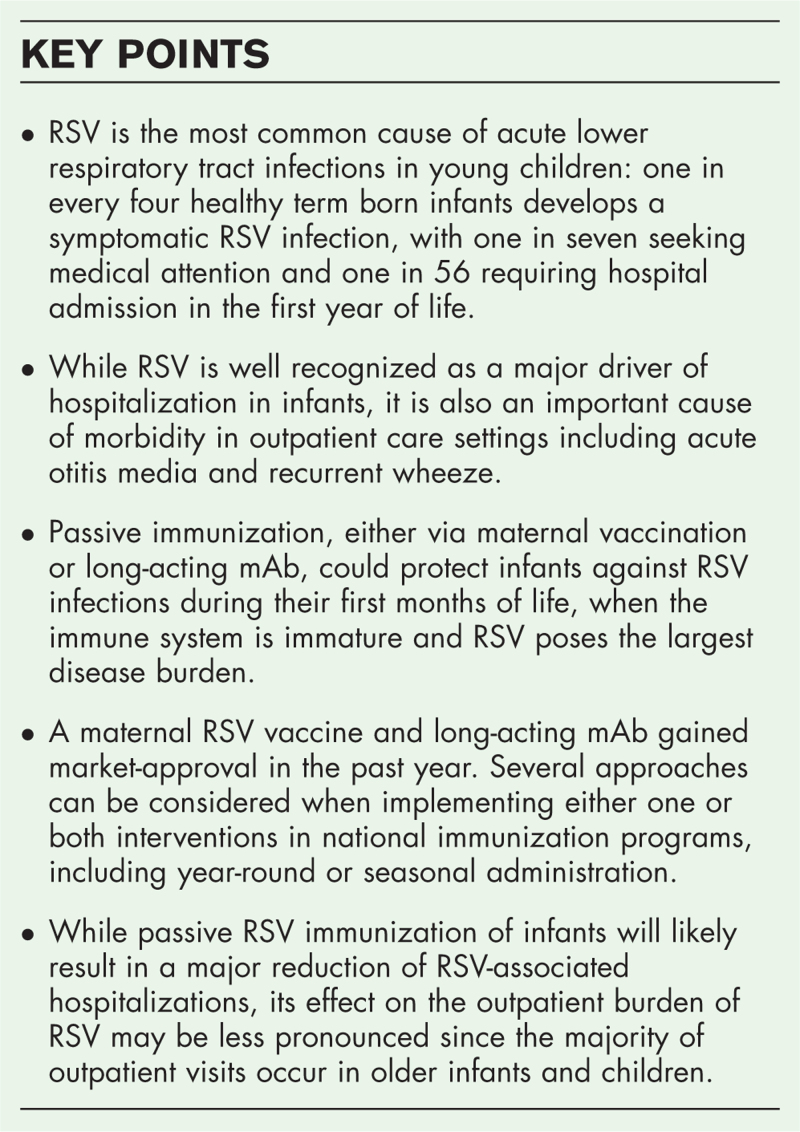
no caption available

With RSV immunization within reach, healthcare professionals, particularly those in primary care, obstetrics, pediatrics and public health, will likely be faced with questions from parents seeking guidance and information (Box 1). The purpose of this review is to provide healthcare professionals in high-income countries with the latest information necessary to adequately inform parents. To this end, we synthesize recent literature on the burden of RSV infection, clinical manifestations, evaluation and management, and the promise of novel preventive interventions, with a special focus on the outpatient setting.

Box 1 **Frequently asked questions (FAQ) by parents to healthcare professionals^a^**

1.
**What is RSV, and what are signs of RSV infection?**
  RSV is a common cause of respiratory infection in children. Symptoms can include a runny nose, coughing, wheezing, fever, and difficulty breathing.
2.
**How common is RSV, and what is the risk for my child to become seriously ill?**
  Nearly all children get infected by RSV before the age of two. Most infections are mild, but breathing difficulties can become severe for some children. During the first year of life, one in seven healthy babies visit a doctor and one in 56 are being hospitalized because of RSV infection. Some children, particularly premature infants and those with underlying health issues, are at higher risk. In high-income countries, mortality due to RSU infection is extremely rare.
3.
**What options are there to prevent RSV infection in my child?**
  Good hand hygiene and avoiding close contact with sick individuals can reduce the risk of RSV infection. Recently, RSV disease can be prevented either by giving antibody products to babies, or by giving their mothers RSV vaccine during pregnancy. For most babies, either the maternal RSV vaccine or the preventive antibody is recommended, but not both.
4.
**How do these RSV immunizations work, and how effective are they?**
  Both immunizations protect against severe RSV disease, but they provide immunity differently. The maternal vaccine triggers the mother's immune system to produce RSV antibodies, which are passed to the baby during pregnancy. Nirsevimab (Beyfortus) is a long-acting monoclonal RSV antibody given directly to the baby. Both immunizations require only a single dose (one shot) and offer over 70% protection against severe RSV for at least five months, but protection fades over time.
5.
**What are potential side effects?**
  Most frequently reported side effects by pregnant people included pain at the injection site, headache, muscle pain, and nausea. Although not common, the clinical trials showed a small imbalance in preterm births (5.7% in vaccinated people vs. 4.7% in those receiving placebo). It is unclear if this is related to the RSV vaccine or if this occurred for reasons unrelated to vaccination; this will be further studied. Side effects after nirsevimab were rare and mainly included pain, redness and swelling at the injection site, and rash.
  ^a^These questions have been formulated in collaboration with parents on the RSV Patient Advisory Board of the ReSViNET foundation.

## THE BURDEN OF RESPIRATORY SYNCTIAL VIRUS

### Global disease burden

A recent review estimated that in 2019 RSV caused 33 million LRTI (22% of all LRTI episodes), 3.6 million hospitalizations, and 101 400 deaths (2% of all deaths) among children less than 5 years globally [1^▪▪^]. Of note, over 97% of RSV-attributable deaths occur in low-income and middle-income countries [8].

### Clinical burden of disease in high-income countries

Although the RSV-attributable mortality in high-income countries is low (typically <0.5% and limited to children with underlying medical conditions) [9], the disease burden remains considerable. Recent data from the Respiratory Syncytial virus Consortium EUrope (RESCEU) prospective birth cohort study including nearly 10 000 healthy term born infants from five European countries, showed that one in four (26.2%) healthy term born infants develops a symptomatic RSV infection, one in seven (14.1%) seeks medical attention, and one in 56 (1.8%) is hospitalized for RSV in the first year of life [10^▪▪^]. Most hospitalizations (60%) occurred in infants younger than three months.

The majority of RSV infections, however, do not require hospitalization and are managed in the community [10^▪▪^,^11^]. Yet, the community burden of RSV is relatively poorly studied, and its true extent may not be fully recognized [9,12]. For example, only 3% of outpatients with confirmed RSV infection were recognized as such, compared with 45% of inpatients according to an U.S. study [13]. A modeling study from the UK estimated that RSV accounts for 11–14% of all primary care visits for respiratory illness among children less than 5 years of age [14]. Among U.S. children less than 5 years, it was estimated that 11–13% visit primary care and 4–6% an emergency department because of RSV infection annually [13]. Noteworthy, in this study, 61% of RSV outpatients were aged between 2 and 5 years, illustrating that a significant proportion of the outpatient RSV burden results from older children. The true burden of RSV however extends beyond the medically attended infections with 49–70% of symptomatic RSV infections managed by parents at home [10^▪▪^,^11^]. A recent observational study including infants from five European countries showed that these infections can cause significant illness with symptom durations similar to those seeking medical attention [15].

### Societal burden

RSV infections are associated with substantial social and economic costs. The global healthcare costs of RSV infections in young children were estimated at €4.82 billion in 2017, with 55% of costs caused by hospitalizations [2]. In the USA, infants’ RSV healthcare costs may be as high as $710 million annually [16].

Yet, the economic impact of RSV encompasses not only direct healthcare costs, but also indirect costs due to reduced work productivity and absence from work among parents. In the USA, caregivers of children with RSV infections miss over 700 000 workdays annually [17], and parental work absenteeism was reported in 52–79% of RSV episodes in Finnish children [18,19]. In the UK, total societal costs due to RSV are projected at £80 million, of which £14 million are due to productivity loss by caregivers, and approximately half the costs are incurred by children less than 1 year [20].

RSV infections also negatively affect quality of life of infected children and their families, even when medical attendance is not required [15,21,22].

## VIROLOGY, SEASONALITY AND TRANSMISSION

RSV is an enveloped, single-stranded RNA virus that belongs to the *Pneumoviridae* family. The virus has two important surface glycoproteins: the G (attachment) protein enables RSV to bind to host cells, and the F (fusion) protein allows fusion of virus and host membrane. The prefusion state of the F protein contains a major antigenic site, which serves an effective target for neutralizing antibodies [23]. RSV is often classified into an A and B subtype, yet there are no major differences in disease severity [24]. The two subtypes can co-circulate during RSV epidemic season every year. Most countries have seasonal RSV outbreaks during the winter, while subtropical areas may show a more endemic pattern [25]. RSV is transmitted through inoculation of air droplets and aerosols produced by infected individuals, but the virus can remain viable on hard surfaces for up to 6 h [26]. The incubation period is usually between 3 and 6 days [27].

## PATHOPHYSIOLOGY

RSV primarily infects epithelial cells in the nasopharynx, leading to destruction of ciliated epithelial cells lining the airways, and the characteristic formation of syncytia (hence the name of the virus). The infection can remain confined to the upper respiratory tract, but may rapidly spread towards the lower airways, reaching the terminal bronchioles. Here, the acute inflammatory responses upon viral replication can trigger capillary leakage, interstitial swelling, inhibited pulmonary surfactant function and excessive mucus production [28]. This, in turn, may lead to severe respiratory symptoms due to airway obstruction and air trapping, especially within the still-narrow bronchioles of infants.

## CLINICAL MANIFESTATIONS AND DIAGNOSIS

A first RSV infection in early childhood is usually symptomatic [29]. Symptoms may range from mild to potentially life-threatening. Most RSV infections cause typical ‘common cold’ symptoms such as rhinitis and nasal congestion. Fever is present in 48–56% [30], while acute otitis media (AOM) may occur in up to 50–77% of young children with RSV infection [18,31–33].

LRTI occurs in 15–50% of young children with RSV infection [34], most notably bronchiolitis (<24 months) but pneumonia and croup are also seen. The diagnosis of bronchiolitis is clinical. Typically, signs and symptoms begin with rhinitis and cough, which over the course of 2–5 days progress to wheeze, rales and breathing difficulty, often accompanied by feeding problems and low-grade fever [30,35]. Diffuse inspiratory crackles and expiratory wheezing may be heard upon auscultation. Respiratory distress can vary from minimal to life-threatening. Usually, symptoms peak around day 5 of the illness and improve by day 7–10, yet cough can persist for up to 3–4 weeks [36].

Infants, particularly those less than 1 months of age or born prematurely, may also develop potentially life-threatening apnea. Of note, apnea can be the first clinical manifestation of RSV infection, and may occur regardless of the severity of other RSV-related symptoms [37].

While other viral pathogens like influenza and rhinovirus can also cause bronchiolitis, RSV accounts for approximately 60–80% of episodes [9,38]. Point-of-care viral testing is becoming increasingly available in the outpatient setting. However, given that specific RSV diagnosis has no clinical implications and does not exclude the possibility of coinfection, its clinical utility is currently limited, and warrants further evaluation.

## CLINICAL EVALUATION AND MANAGEMENT IN THE OUTPATIENT SETTING

### Evaluation

The outpatient evaluation and management of a young child with bronchiolitis is challenging since it is difficult to predict which child will develop severe disease. Most infants have mild disease, which can be managed at home; approximately 5–10% of those attending primary care will require hospitalization, of which 2–6% require intensive care admission [10^▪▪^,^13^,^39^].

Risk factors for severe RSV disease include prematurity, chronic lung disease, congenital heart disease, Down syndrome, neuromuscular disease and immunodeficiency (Box 2) [9,40]. However, the majority of infants hospitalized with RSV are born term and have no underlying medical conditions [13]. In these children, young age (<6 months) is the most important risk factor [10^▪▪^].

Box 2 **Risk factors and red flags for severe RSV infection**

 - Age <6 months

 - Prematurity

 - Chronic lung disease

 - Hemodynamically significant congenital heart disease

 - Down syndrome

 - Neuromuscular disease

 - Immunodeficiency

 - Poor nutritional status

 - In-utero or household smoke exposure

**Red flags from history and examination in outpatient setting**

Immediately refer for emergency hospital care in case of any of the following:

 - Apnea, defined as prolonged breathing stop (at least 10 s in infancy)

 - Signs of severe respiratory distress, such as grunting, marked scalene or costal chest retractions, increased respiratory rate of over 70 breaths/min (count over the course of at least one minute)

 - Central cyanosis

 - Infant of child looks seriously unwell to healthcare professional

 - Persistent oxygen saturation of less than 92%, when breathing room air

Refer to pediatrician/hospital in case of any of the following:

 - Altered mental status, such as lethargy, irritability, excessive sleepiness

 - Poor feeding (<50-75% of usual volume) with signs of dehydration, such as decreased urine output and tearless crying

Consider referring to a hospital in case of any of the following:

 - Age <1 month

 - Respiratory rate of over 60 breaths/min

Medical history and examination should be primarily focused on evaluating adequate breathing and hydration. The presence of signs of serious illness (Box 2) requires prompt hospital referral for further evaluation and supportive care. Several scoring tools are available that can be helpful for assessing respiratory difficulty in infancy, yet only the ReSViNet Scale has been validated for use in primary care [41]. Pulse oximetry should only be used if validated age-appropriate devices are available. Clinicians should be aware that clinical findings can change rapidly due to the airways alternately getting clogged and cleared of mucus and debris, particularly in young infants. This fluctuation can make assessment tricky. Therefore, parents should always be provided with a safety netting advice to reconsult if symptoms worsen rapidly or significantly (Box 2).

### Secondary bacterial infections

RSV infection may be complicated by the development of a secondary bacterial infection (SBI). The reported incidence of SBI is highly variable and depends on the methods used [42]. Among children hospitalized with RSV, estimates range from 1.2 to 44% [43]. In the outpatient setting, the incidence of SBI is unknown, yet the risk is generally thought to be low, even if infants are febrile [44]. Due to the lack of a valid reference test, the diagnosis of an SBI is challenging. Diagnostic tools such as chest X-ray, laboratory and microbiology tests vary in availability, cost and utility in outpatient settings, and are generally unhelpful in clinical management [45]. Therefore, current guidelines emphasize that those diagnostic modalities should not be used routinely [46].

### Treatment modalities

Table 1 summarizes over an overview of treatment recommendations for RSV bronchiolitis in the outpatient setting. Supportive care is the mainstay of clinical management. In short, the routine use of bronchodilators (B2-agonists and epinephrine), inhaled and systemic corticosteroids, leukotriene antagonists and antibiotics is not recommended [46–50]. Antibiotics and inhaled corticosteroids are also not recommended to prevent persistent wheeze or cough following bronchiolitis [51,52].

**Table 1:** Treatment recommendations for RSV bronchiolitis in the outpatient setting

Medicine		
Antibiotics	Not recommended	Antibiotics have no effect on the course of acute bronchiolitis [92], nor do they prevent persistent respiratory symptoms in the postacute bronchiolitis phase [51]. Antibiotics should only be used in case of concurrent bacterial infection.
Bronchodilators	Not recommended	Bronchodilators do not reduce the need for hospitalization nor shorten the length of illness [93].
Inhaled steroids	Not recommended	Inhaled corticosteroids have no effect on the course of acute bronchiolitis, nor are they effective in preventing recurrent wheeze [52,94]
Epinephrine	Not routinely recommended	Nebulised epinephrine may have a modest effect among outpatients on the risk of hospital admission in the early stages of disease [95]
Systemic steroids	Not recommended	Systemic corticosteroids have no or minimal effect on the course of acute bronchiolitis [94]
Chest physiotherapy	Not routinely recommended	There is low-certainty evidence that chest physiotherapy may result in mild to moderate improvement of disease severity for those with moderately severe bronchiolitis in outpatient settings [96]
Hypertonic saline	Not routinely recommended	Nebulised hypertonic saline may slightly reduce the risk of hospitalization amongst outpatients [97]
RSV-specific monoclonal antibodies	Not recommended	RSV monoclonal antibodies should not be used to treat acute viral bronchiolitis. They are used for RSV prevention.
Leukotriene receptor agonists	Not recommended	Montelukast has no effect on the course of acute bronchiolitis nor on postbronchiolitis wheeze [98]

Antipyretics can help ease discomfort, and since infants primarily breath through their noses, saline nasal drops can provide significant relief from breathing/feeding difficulties caused by nasal congestion.

Antibiotic use should be reserved for strongly suspected or confirmed SBI and selected cases of AOM (e.g. bilateral AOM <2 years, AOM and acute ear discharge, and specific at-risk groups) [46,53]. Although SBIs are considered rare in the outpatient settings, antibiotics were prescribed to nearly 20% of outpatients less than 2 years with bronchiolitis in the USA [54], and up to 71% of RSV outpatients less than 1 year in Finland [33], suggesting substantial overuse. According to a population-based modeling study, RSV may be responsible for nearly 7% of all outpatient antibiotic prescriptions in children below 5 years of age in Scotland [55].

Currently, ribavirin is the only antiviral therapy approved for treating RSV, but its poor benefit-safety profile and high costs limit its use in practice [56,57]. Hence, ribavirin should only be considered for treating life-threatening RSV infections in immunocompromised patients. There are several other antiviral drugs in clinical development [58]. For ziresovir, a first oral fusion inhibitor to treat RSV positive phase III trial results was recently announced [59,60]. Although promising, more trial data are needed before considering the use of antivirals for RSV infections in everyday practice.

## LONG-TERM IMPACT: THE LINK BETWEEN RESPIRATORY SYNCTIAL VIRUS AND WHEEZING/ASTHMA

Children who experienced severe RSV infection in early life often develop recurrent wheezing, and are at an increased risk of developing childhood asthma [61^▪▪^,^62^,^63^]. However, whether RSV infection is causally related to recurrent wheezing/asthma, or rather a marker of shared genetic predisposition, has been a subject of longstanding debate [62,64].

Findings from a recent U.S. prospective birth cohort study strengthen the case for a potential causal link. Unlike most previous studies that focused on severe RSV infections requiring hospitalization, this study used both molecular and postseason serological tests to ensure all RSV infections during infancy were captured, irrespective of severity [61^▪▪^]. At age 5 years, children who were not infected with RSV during infancy had a 26% lower risk of asthma compared to those that were infected. The authors estimated that 15% of childhood asthma cases could be prevented by avoiding RSV infection during infancy.

Whether RSV prevention will indeed reduce recurrent wheeze or asthma is, however, still unclear. Clinical trials of short-acting RSV mAbs in preterm infants showed conflicting results. In the MAKI-trial, including 429 healthy preterm infants, palivizumab reduced recurrent wheeze by nearly 50% in the first year of life [65]. However, no significant impact on asthma or lung function was observed at age 6 years [66]. Similarly, a trial with motavizumab, another short-acting mAb, found reduced parent-reported wheezing but not physician-diagnosed wheezing at age 1–3 years [67]. Yet, these studies are limited to preterm infants and may not be applicable to term born infants. Information for the latter group could be gained from long-term follow-up of recent clinical trials assessing the efficacy of passive RSV immunization in healthy term infants.

## PREVENTION

Given the major burden and limited treatment options, prevention of RSV infections is being considered an urgent global health priority [68]. Since RSV is transmitted through aerosols, nonpharmacological hygiene measures, such as hand washing and face mask-wearing in case of respiratory symptoms, remain a key strategy for reducing its spread [69].

**FIGURE 1:**
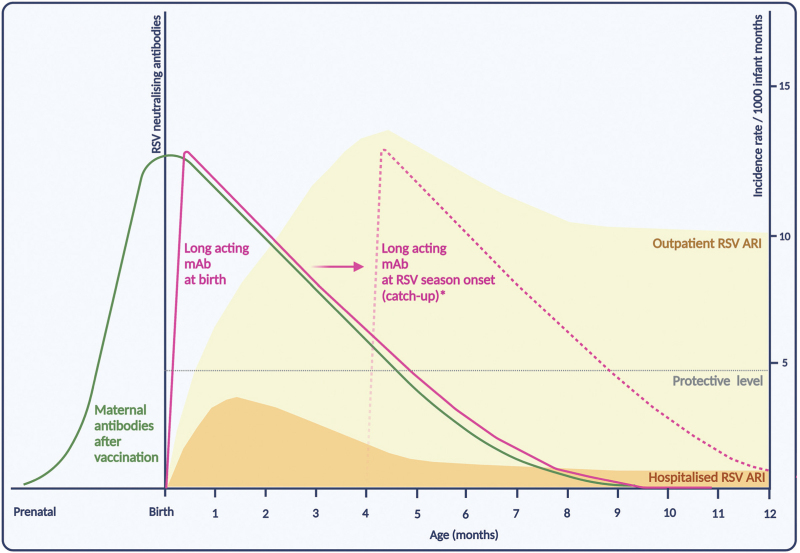
Conceptual representation of infant RSV immunization strategies in relation to the incidence rates of hospitalized and outpatient RSV acute respiratory infections (ARI), by age in months. The incidence rates for hospitalized and outpatient RSV ARI are based on the RESCEU study of healthy term born infants during their first year of life [10^▪▪^]. Please note that the levels of neutralising antibodies for the various immunization strategies are only indicative, assuming ∼5 months protection. This figure was created with Biorender.com. ∗Long acting monoclonal antibodies can be administered at birth for infants born in the RSV season, or at onset of the RSV season for infants born outside the season (potentially at any age).

Thus far, RSV preventive interventions have primarily been aimed at young infants (<6 months). The immature nature of the newborns’ immune system, however, hinders the ability to produce an effective immune response to vaccination in the first weeks of life. Therefore, the primary strategy to protect infants against RSV is passive immunization, either via maternal vaccination or via mAbs (Supplementary Table 1). Although both approaches only provide temporary protection, they do possess the potential to cover the infant's window of critical vulnerability (Fig. 1).

At present, more than 20 mAbs and vaccine candidates are in various stages of clinical trials [70]. Two recent reviews provide a detailed overview of RSV interventions in development [58,71^▪^]. Here, we focus on products that are either already available or expected to be available shortly.

### mAbs

Passive immunization using mAbs is based on the principle that these antibodies hinder RSV from entering cells. Until recently, only one mAb, palivizumab, had been licensed and that occurred over 20 years ago [72]. Its use is preserved to high-risk groups, while most infants hospitalized with RSV are healthy term-born babies, emphasizing the need for a universal prevention strategy.

In 2022, nirsevimab became the first RSV mAb with an extended half-life to gain regulatory approval [6,7]. In healthy term and preterm born infants, a single intramuscular injection with nirsevimab showed an efficacy of 79.5 and 77.3% against medically attended RSV LRTI and RSV hospitalization during 150 days of follow-up, respectively [73^▪▪^]. This covers the length of a typical RSV season. No safety concerns were identified, and side effects were rare and generally mild, such as local injection-site pain or swelling [74]. Also, nirsevimab is not expected to interfere with the immune response to other vaccines, and can be concomitantly administered with other routine childhood vaccines [75].

Another long-acting mAb, clesrovimab, showed promising results in a phase II trial [76], and is currently being studied in a phase III trial [77]. In the future, implementation of multiple mAbs might be considered to counter the possible emergence of escape mutations.

### Maternal immunization

Another promising approach for infant RSV prevention is maternal immunization. Maternal antibodies produced upon active RSV immunization during pregnancy are transferred to the fetus via the placenta, providing protection in the first months of life [78]. A limitation of maternal vaccination is the reduced benefit for infants born prematurely, since antibody transfer reaches peak levels only towards the end of the third trimester.

In 2023, the FDA and EMA approved a first maternal RSV vaccine [4,5]. In a worldwide phase III trial, this maternal vaccine was associated with 81.8 and 70.9% reduction in medically attended severe LRTI within 90 and 150 days after birth, respectively [79^▪▪^]. Safety concerns, however, have been raised due to a minimal and not statistically significant increase in premature births in the vaccinated group. A trial with another maternal vaccine was halted in 2022 because of a significant increase in preterm births [80]. EMA considered the maternal RSV vaccine well tolerated after gestational age 24 weeks, whereas the FDA only approves administration relatively late in pregnancy, during weeks 32 through 36, as precaution against possible preterm birth [81].

### Implementation strategies and their challenges

Either one or both passive immunizations are likely to be introduced, or have already been implemented, in national immunization programs. Several immunization strategies can be considered, including year-round and seasonal approaches (Supplementary Table 2). While a seasonal approach provides optimal protective titers during the RSV season (Supplementary Figure 1), a year-round approach may be easier to implement.

Since universal use of mAbs for infant immunization would be a novelty, its introduction would require clear counseling of parents to address any potential hesitancy towards mAb use. In theory, with maternal vaccines for influenza and pertussis already in use, maternal RSV immunization could be more straightforward. However, in those countries with poor maternal vaccine coverage, implementation of this novel intervention may pose a challenge [82]. Implementing a combined strategy of maternal RSV immunization and mAbs for those born prematurely might pose challenges in terms of execution.

The preferred strategy will likely differ across countries, depending on factors like RSV seasonality, costs, cultural preferences and healthcare system organization. France and Spain have already adopted a seasonal mAb program for the 2023–2024 RSV season [83,84], whereas the UK favors a year-round program (either mAb or maternal vaccination) for ease of logistics [85]. In the USA, the Centers for Disease Control and Prevention (CDC) recommends the seasonal use of either nirsevimab or maternal RSV vaccination without a distinct preference for one over the other [86].

### Respiratory syncytial virus prevention in older children (>12 months)

Even though infants face the highest risk for severe infections, RSV poses a substantial burden beyond infancy. For older aged children, active immunization may be a more promising approach by providing sustained protection against RSV throughout childhood. Additionally, this approach may curtail RSV transmission in daycare facilities and (pre)schools, thereby reducing the risk of introducing RSV into households with younger, more vulnerable siblings. Several vaccine candidates, targeted at the pediatric population, are currently in early stages of clinical development [70].

## ANTICIPATED IMPACT OF INFANT RESPIRATORY SYNCTIAL VIRUS IMMUNIZATION

Immunizing infants against RSV is expected to substantially reduce the burden of RSV in the first year of life, with total impact varying by country depending on the immunization strategy, vaccine uptake, and RSV incidence. For example, assuming a 71–80% vaccine uptake, seasonal immunization with nirsevimab may reduce RSV-related hospital admissions in the USA by 53%, emergency department visits by 55%, and primary care visits by 55% for infants in the first year of life [87]. RSV immunization is likely to also reduce RSV-associated morbidity and antibiotic use among infants. In a phase III trial, maternal RSV immunization reduced antibiotic prescriptions in infancy by 12.9% over the first 3 months of life [88].

The effect of infant RSV immunization will likely be less pronounced in the outpatient than the hospital setting. While most RSV-associated hospitalizations occur in young infants (<6 months), the majority of RSV outpatient visits occur in older infants and children [10^▪▪^,^13^] (Fig. 1).

Moreover, as both mAb and maternal immunization only offer transient protection, these strategies merely postpone a child's first RSV infection to their second RSV season. It remains uncertain whether this deferred infection might lead to more severe clinical symptoms at relatively older age, similar to what was observed after the COVID-19 pandemic; recent data from Denmark and the Netherlands showed an increase in hospital admissions among older children during the resurgence of RSV following relaxation of COVID-19 restrictions [89].

Also, the potential impact of RSV immunization on other respiratory virus infections remains to be elucidated. Multiple viruses often co-circulate, potentially infecting the respiratory tract either at the same time or sequentially, a phenomenon called ‘viral interference’. Interestingly, it has been observed that RSV might block or suppress infection by other viruses when simultaneously present in the same host, which implies that any prevented RSV infection might be replaced by other viral infections such as rhinovirus [90]. On the contrary, co-infection by RSV and human metapneumovirus is reported to result in more severe disease, which would suggest a beneficial effect of RSV prevention [91].

## CONCLUSION

Childhood RSV infections pose a major public health burden. This burden extends beyond the health challenges faced by infected children to emotional and practical burden on affected families, and puts a substantial strain on the healthcare system and economy. Encouragingly, two distinct preventive interventions have been registered in the USA and Europe for protecting infants against RSV in the first months of life. Both interventions are well tolerated and effective, and parents should be counseled accordingly for successful uptake. Awaiting pediatric vaccines, RSV will continue to be a common reason for outpatient visits in older children (>12 months).

## Acknowledgements

*None.*

### Financial support and sponsorship

*None.*

### Conflicts of interest

*S.H. and R.P.V. report no potential conflicts.*

*J.G.W. has been an investigator for clinical trials sponsored by pharmaceutical companies, including AstraZeneca, Merck, Pfizer, Sanofi and Janssen. All funds have been paid to UMCU. J.G.W. participated in the advisory board of Janssen and Sanofi with fees paid to UMCU.*

*L.J.B. has regular interaction with pharmaceutical and other industrial partners. He has not received personal fees or other personal benefits. UMCU has received major funding (>€100000 per industrial partner) for investigator-initiated studies from AbbVie, MedImmune, AstraZeneca, Sanofi, Janssen, Pfizer, MSD, and MeMed Diagnostics. UMCU has received major funding for the RSV GOLD study from the Bill & Melinda Gates Foundation. UMCU has received major funding as part of the public private partnership IMI-funded RESCEU and PROMISE projects with partners GSK, Novavax, Janssen, AstraZeneca, Pfizer and Sanofi. UMCU has received major funding from Julius Clinical for participating in clinical studies sponsored by MedImmune and Pfizer. UMCU received minor funding (€1000–25000 per industrial partner) for consultation and invited lectures by AbbVie, MedImmune, Ablynx, Bavaria Nordic, MabXience, GSK, Novavax, Pfizer, Moderna, AstraZeneca, MSD, Sanofi, Genzyme and Janssen. L.J.B. is the founding chairman of the ReSViNET Foundation.*

## Supplementary Material

Supplemental Digital Content

## REFERENCES AND RECOMMENDED READING

Papers of particular interest, published within the annual period of review, have been highlighted as:

▪ of special interest
▪▪ of outstanding interest
