# Supplementary material for: Outpatient respiratory syncytial virus infections and novel preventive interventions
Source: Curr Opin Pediatr. 2023 Dec 12;36(2):171–81. doi: 10.1097/MOP.0000000000001323 (PMC10919273; doi:10.1097/MOP.0000000000001323)
Supplement: Supplemental Digital Content [file coped-36-171-s001.docx]

**
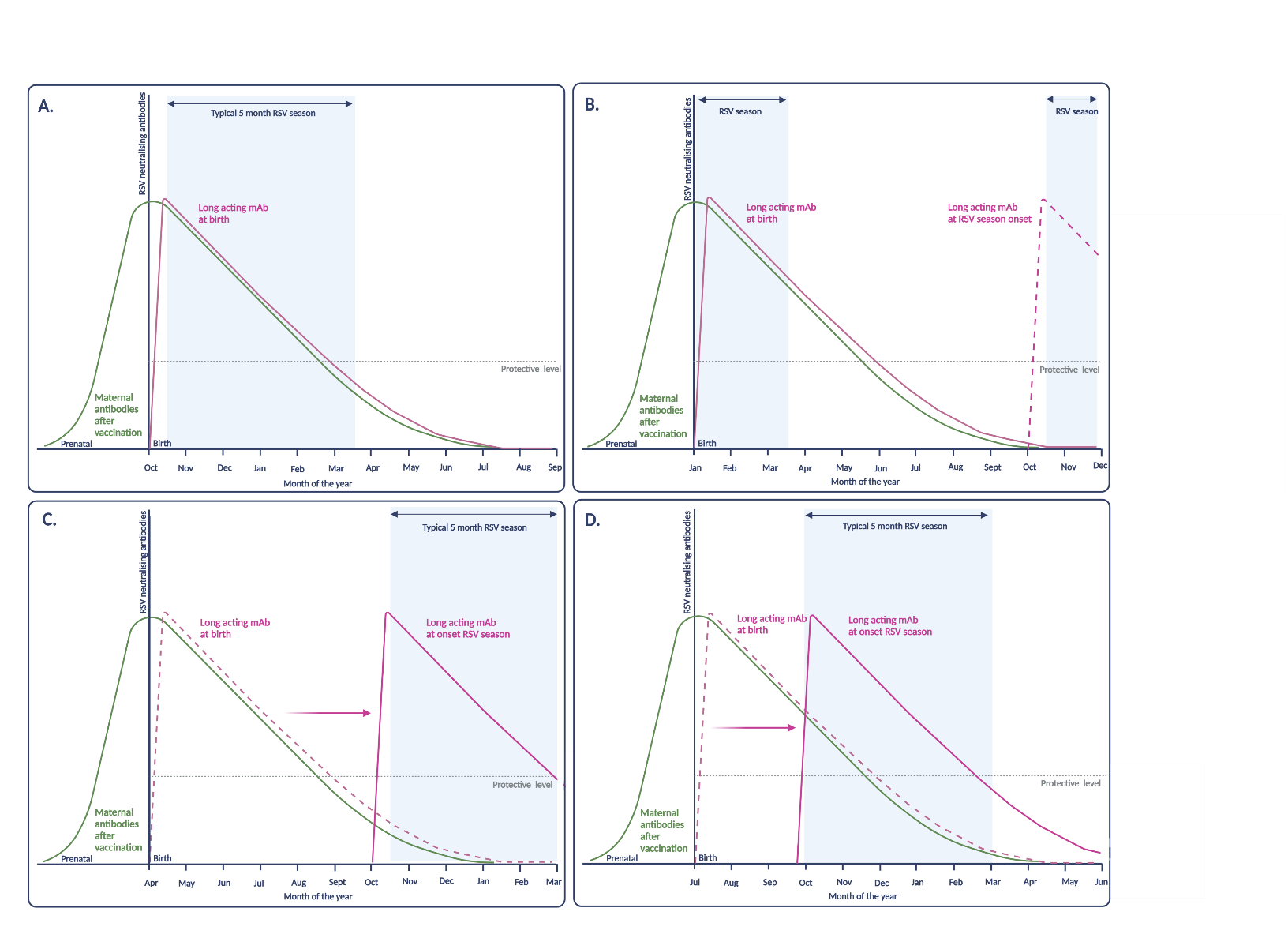
**

**Supplementary Figure 1.** Conceptual overview of timing of protection for different RSV immunization strategies in term infants born in: **A) October; B) January; C) April; D) July,** while assuming an RSV season of 5 months between October and March. Please note that RSV seasonality differs per region and per year, and that levels of neutralizing antibodies are only indicative, assuming ~5 months protection.

**Supplementary Table 1. Characteristics of long-acting monoclonal antibodies and maternal vaccine**

|  | Long-acting monoclonal antibodies | Maternal immunization |
| --- | --- | --- |
| Mechanism | Immunoprophylaxis | Transplacental transfer of maternal IgG antibodies |
| Administration | Intramuscular (infant), once | Intramuscular (mother), once |
| Timing of administration | At birth (year-round), or at start of RSV season (seasonal) | During 2^nd^ or 3^th^ trimester of pregnancy |
| Onset of protection | Immediately after administration | Immediately after birth |
| Name of product and its efficacy in phase III trial | Nirsevimab  (Beyfortus™)^1^  During 150 days after administration:  Medically attended RSV LRTI:  79.5% (95%-CI: 65.9-87.7)  RSV-associated hospitalization:  77.3% (95%-CI: 50.3-89.7) | Bivalent pre-F maternal vaccine (Abrysvo™)^2^  During 90 days after birth  Medically attended RSV LRTI:  57.1% (99.5%-CI: 14.7-79.8)  RSV-associated hospitalization:  67.7% (99.2%-CI: 15.9-89.5)  Severe RSV LRTI:  81.8% (99.5%-BI: 40.6-96.3)  During 150 days after birth:  Medically attended RSV LRTI:  52.5% (99.5%-CI: 28.7-68.9)  Severe RSV LRTI:  70.9% (99.5%-CI: 44.5-85.9) |
| Considerations |  | Possibly less effective in the case of premature birth or a mother with weakened immunity |

LRTI: lower respiratory tract infection.

References:

1. Simões EAF, Madhi SA, Muller WJ, et al. Efficacy of nirsevimab against respiratory syncytial virus lower respiratory tract infections in preterm and term infants, and pharmacokinetic extrapolation to infants with congenital heart disease and chronic lung disease: a pooled analysis of randomised controlled trials. *Lancet Child Adolesc Health*. 2023;7:180-189. doi:10.1016/S2352-4642(22)00321-2
2. Kampmann B, Madhi SA, Munjal I, et al. Bivalent Prefusion F Vaccine in Pregnancy to Prevent RSV Illness in Infants. *N Engl J Med*. 2023;388(16):1451-1464. doi:10.1056/NEJMoa2216480

**Supplementary Table 2. Potential strategies for infant RSV immunization**

| Year-round strategies | |
| --- | --- |
| Year-round long-acting mAb | Year-round mAb administration to all infants shortly after birth, regardless of birth month |
| Year-round maternal vaccine + long-acting mAb for premature infants | Year-round administration of maternal vaccine during pregnancy, complemented by mAb administration to those infants that are born premature |
| Seasonal strategies | |
| Seasonal long-acting mAb | mAb administration to all infants born during the RSV season |
| Seasonal long-acting mAb + catch-up | mAb administration to all infants born during the RSV season, and at the onset of the RSV season to all infants born outside of the season (catch-up) |
| Seasonal maternal vaccine + long-acting mAb for premature infants | Seasonal administration of maternal vaccine during pregnancy to mothers expected to deliver just before or during the RSV season, complemented by mAb administration to those infants that are born premature |

mAb; monoclonal antibody.
